# Supplementary figures and images for: Sub-lethal effects of the consumption of Eupatorium buniifolium essential oil in honeybees
Source: PLoS One. 2020 Nov 4;15(11):e0241666. doi: 10.1371/journal.pone.0241666 (PMC7641371; doi:10.1371/journal.pone.0241666)

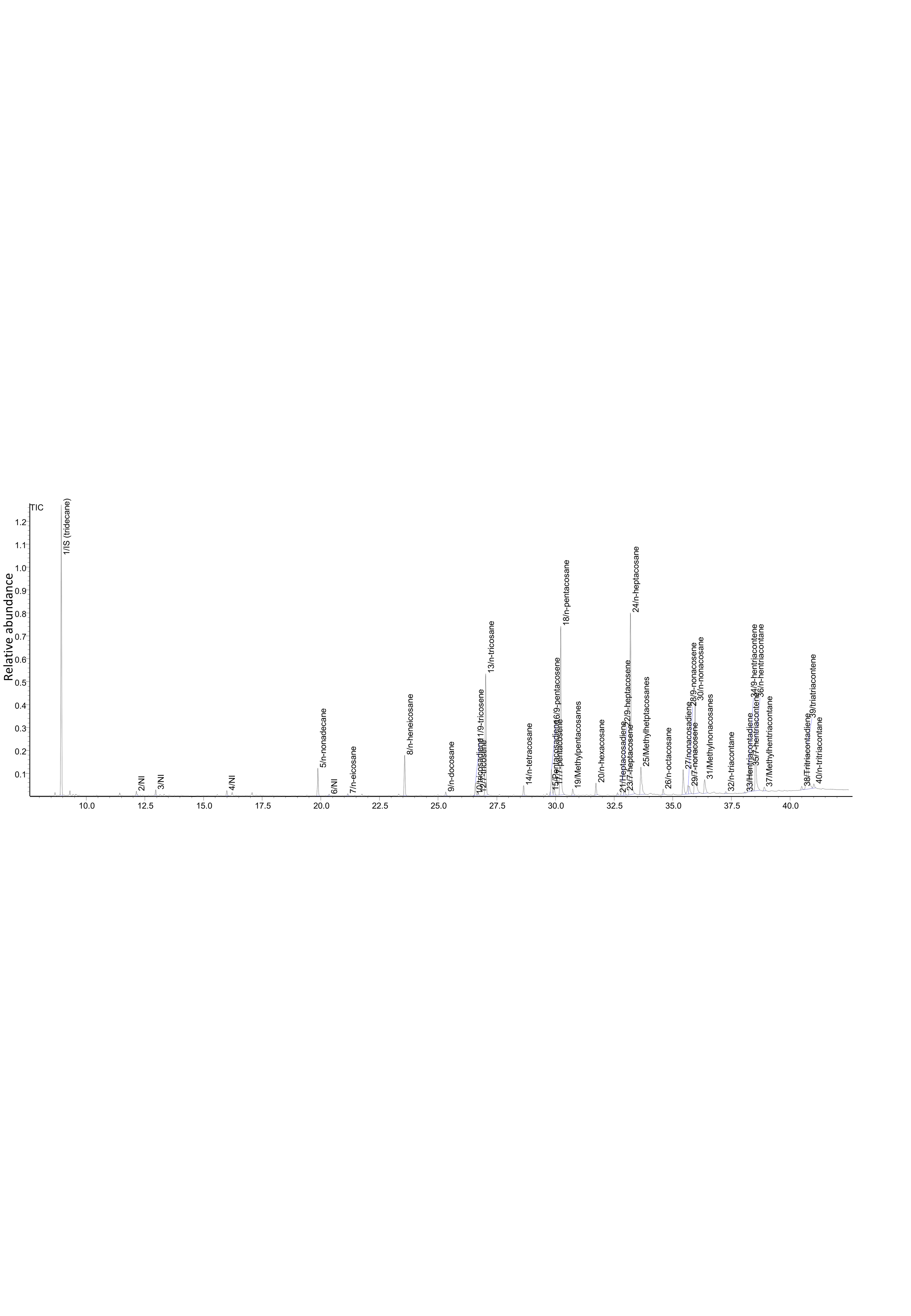

Supplement: S1 Fig — Numbers of peaks are as in S1 and S2 Tables. (TIF) [file pone.0241666.s001.tif]
