# Supplementary material for: Sub-lethal effects of the consumption of Eupatorium buniifolium essential oil in honeybees
Source: PLoS One. 2020 Nov 4;15(11):e0241666. doi: 10.1371/journal.pone.0241666 (PMC7641371; doi:10.1371/journal.pone.0241666)
Supplement: S1 Table — Results are shown as mean ± se (N = 4 per treatment). (DOCX) [file pone.0241666.s002.docx]

S1 Table: Amount of CHC in honeybees fed on diets enriched with EOEb (2 EOEb were supplied: EOEb-2009 and EOEb-2014) in 3 concentrations (300, 3000 and 6000 ppm; Experiment I). Results are shown as mean ± se (N = 4 per treatment).

|  | |  | |  | | **Amount of CHC (μg/bee)** | | | | | | | |
| --- | --- | --- | --- | --- | --- | --- | --- | --- | --- | --- | --- | --- | --- |
| **Peak N^o^** | **Compound ID** | **Compound group** | **MW** | **Retention Time** | **Retention index** | **Control** | **Control (Ethanol)** | **EOEb_2014_300** | **EOEb_2014_3000** | **EOEb_2014_6000** | **EOEb_2009_300** | **EOEb_2009_3000** | **EOEb_2009_6000** |
| 2 | NI^ξ^ | UK^ⱡ^ |  | 12.138 | 1476 | 1.8 ± 0.4 | 2.1 ± 0.4 | 3.8 ± 0.4 | 4.3 ± 0.7 | 9.6 ± 0.6 | 1.5 ± 0.1 | 3.9 ± 0.6 | 4.4 ± 0.4 |
| 3 | NI | UK |  | 12.96 | 1521 | 0.13 ± 0.01 | 0.12 ± 0.01 | 0.18 ± 0.02 | 0.08 ± 0.01 | 0.07 ± 0.01 | 0.087 ± 0.001 | 0.14 ± 0.01 | 0.1 ± 0.01 |
| 4 | NI | UK |  | 15.994 | 1687 | 0.1 ± 0.03 | 0.08 ± 0.01 | 0.12 ± 0.02 | 0.1 ± 0.03 | 0.13 ± 0.01 | 0.06 ± 0.02 | 0.1 ± 0.03 | 0.15 ± 0.01 |
| 5 | n-nonadecane | alkane | 268 | 19.874 | 1900 | 0.53 ± 0.14 | 0.42 ± 0.06 | 0.55 ± 0.06 | 0.43 ± 0.13 | 0.67 ± 0.1 | 0.31 ± 0.1 | 0.51 ± 0.17 | 0.68 ± 0.03 |
| 6 | NI | UK |  | 20.351 | 1937 | 0.03 ± 0 | 0.022 ± 0.001 | 0.031 ± 0.004 | 0.022 ± 0.002 | 0.022 ± 0.001 | 0.019 ± 0.001 | 0.025 ± 0.003 | 0.03 ± 0.002 |
| 7 | n-eicosane | alkane | 282 | 21.147 | 2000 | 0.06 ± 0.01 | 0.045 ± 0.003 | 0.07 ± 0.01 | 0.037 ± 0.002 | 0.037 ± 0.002 | 0.0392 ± 0.0002 | 0.05 ± 0 | 0.04 ± 0.01 |
| 8 | n-heneicosane | alkane | 296 | 23.572 | 2100 | 4.2 ± 0.3 | 5 ± 1 | 4.1 ± 0.8 | 3.5 ± 0.9 | 6.7 ± 0.9 | 3.3 ± 1.1 | 5.5 ± 1.3 | 5.3 ± 0.2 |
| 9 | n-docosane | alkane | 310 | 25.324 | 2200 | 0.25 ± 0.01 | 0.27 ± 0.03 | 0.2 ± 0.1 | 0.28 ± 0.04 | 0.46 ± 0.04 | 0.21 ± 0.04 | 0.27 ± 0.03 | 0.394 ± 0.004 |
| 10 | tricosadiene | alkadiene | 306 | 26.542 | 2272 | 0.05 ± 0.02 | 0.048 ± 0.004 | 0.06 ± 0.02 | 0.054 ± 0.005 | 0.1 ± 0.03 | 0.06 ± 0.02 | 0.05 ± 0 | 0.1 ± 0.02 |
| 11 | 9-tricosene | alkene | 308 | 26.589 | 2274 | 1.3 ± 0.1 | 1.8 ± 0.1 | 1.5 ± 0.4 | 2.4 ± 0.2 | 3.4 ± 0.4 | 1.6 ± 0.2 | 1.7 ± 0.2 | 3.02 ± 0.04 |
| 12 | 7-tricosene | alkene | 308 | 26.704 | 2281 | 0.8 ± 0.6 | 0.28 ± 0.04 | 0.19 ± 0.05 | 0.39 ± 0.05 | 0.5 ± 0.1 | 0.229 ± 0.048 | 0.2 ± 0.04 | 0.7 ± 0.1 |
| 13 | n-tricosane | alkane | 324 | 27.023 | 2300 | 15.5 ± 1.2 | 18.7 ± 1.4 | 14 ± 3 | 21 ± 1 | 28 ± 2 | 17 ± 2 | 15 ± 3 | 24.3 ± 0.3 |
| 14 | n-tetracosane | alkane | 338 | 28.643 | 2400 | 0.7 ± 0.1 | 0.7 ± 0.1 | 0.6 ± 0.1 | 0.8 ± 0.1 | 1.2 ± 0.1 | 0.7 ± 0.1 | 0.6 ± 0.1 | 1 ± 0.1 |
| 15 | Pentacosadiene | alkadiene | 334 | 29.775 | 2472 | 0.2 ± 0.1 | 0.22 ± 0.02 | 0.1 ± 0.1 | 0.36 ± 0.04 | 0.04 ± 0.01 | 0.3 ± 0.1 | 0.35 ± 0.03 | 0.35 ± 0.03 |
| 16 | 9-pentacosene | alkene | 336 | 29.831 | 2475 | 2.2 ± 0.3 | 2.79 ± 0.04 | 1.9 ± 0.6 | 3.7 ± 0.2 | 5.7 ± 0.8 | 2.6 ± 0.4 | 2.7 ± 0.4 | 4 ± 0.3 |
| 17 | 7-pentacosene | alkene | 336 | 29.945 | 2482 | 0.5 ± 0.1 | 0.6 ± 0.1 | 0.4 ± 0.1 | 1.02 ± 0.04 | 1.3 ± 0.2 | 0.7 ± 0.1 | 0.6 ± 0.1 | 1.1 ± 0.1 |
| 18 | n-pentacosane | alkane | 352 | 30.223 | 2500 | 18.9 ± 2.3 | 24.9 ± 0.9 | 17 ± 4 | 25 ± 1 | 32 ± 2 | 24 ± 2 | 19 ± 3 | 27 ± 2 |
| 19 | Methylpentacosanes | branched alkane | 352 | 30.736 | 2534 | 0.19 ± 0.03 | 0.2 ± 0 | 0.1 ± 0.1 | 0.3 ± 0.1 | 0.44 ± 0.016 | 0.2 ± 0.1 | 0.25 ± 0.03 | 0.314 ± 0.002 |
| 20 | n-hexacosane | alkane | 366 | 31.724 | 2600 | 0.7 ± 0.1 | 0.7 ± 0.1 | 0.7 ± 0.2 | 0.8 ± 0.1 | 1.1 ± 0.1 | 0.8 ± 0.1 | 0.7 ± 0.1 | 1.1 ± 0.2 |
| 21 | Heptacosadiene | alkadiene | 362 | 32.643 | 2662 | 0.014 ± 0.003 | 0.01 ± 0.01 | 0.01 ± 0.002 | 0.005 ± 0.002 | 0.009 ± 0.002 | 0.009 ± 0.003 | 0.012 ± 0.005 | 0.0029 ± 0.0001 |
| 22 | 9-heptacosene | alkene | 364 | 32.844 | 2676 | 1.3 ± 0.2 | 1.6 ± 0.2 | 1.4 ± 0.5 | 2.3 ± 0.2 | 3.2 ± 0.5 | 1.8 ± 0.2 | 1.5 ± 0.3 | 2.5 ± 0.3 |
| 23 | 7-heptacosene | alkene | 364 | 32.955 | 2684 | 0.6 ± 0.1 | 0.7 ± 0.1 | 0.5 ± 0.2 | 0.9 ± 0.1 | 1.2 ± 0.2 | 0.8 ± 0.1 | 0.6 ± 0.2 | 0.92 ± 0.01 |
| 24 | n-heptacosane | alkane | 380 | 33.195 | 2700 | 17.7 ± 2 | 19.4 ± 1.7 | 17 ± 3 | 20 ± 2 | 24 ± 1 | 21 ± 2 | 17 ± 2 | 23 ± 3 |
| 25 | Methylhetptacosanes | branched alkane | 380 | 33.641 | 2732 | 1 ± 0.2 | 1 ± 0.1 | 1 ± 0.2 | 1.2 ± 0.3 | 2.1 ± 0.1 | 1.2 ± 0.3 | 1.4 ± 0.2 | 1.66 ± 0.04 |
| 26 | n-octacosane | alkane | 394 | 34.586 | 2800 | 0.16 ± 0.02 | 0.13 ± 0.01 | 0.16 ± 0.04 | 0.15 ± 0.03 | 0.26 ± 0.02 | 0.16 ± 0.02 | 0.2 ± 0.1 | 0.3 ± 0.03 |
| 27 | nonacosadiene | alkadiene | 390 | 35.444 | 2863 | 0.08 ± 0.05 | 0.09 ± 0.03 | 0.1 ± 0.04 | 0.06 ± 0.03 | 0.14 ± 0.05 | 0.05 ± 0.03 | 0.3 ± 0.1 | 0.14 ± 0.01 |
| 28 | 9-nonacosene | alkene | 406 | 35.652 | 2878 | 0.5 ± 0.1 | 0.5 ± 0.1 | 0.6 ± 0.3 | 0.7 ± 0.1 | 1 ± 0.1 | 0.6 ± 0.1 | 0.7 ± 0.1 | 0.9 ± 0.1 |
| 29 | 7-nonacosene | alkene | 406 | 35.708 | 2882 | 0.6 ± 0.1 | 0.4 ± 0.1 | 0.4 ± 0.1 | 0.4 ± 0.1 | 0.7 ± 0.05 | 0.5 ± 0.1 | 0.7 ± 0.2 | 0.73 ± 0.01 |
| 30 | n-nonacosane | alkane | 408 | 35.952 | 2900 | 4.6 ± 0.5 | 3.5 ± 0.4 | 3.8 ± 0.7 | 3.2 ± 0.5 | 5.1 ± 0.1 | 3.9 ± 0.3 | 5.5 ± 1.3 | 5.3 ± 0.5 |
| 31 | Methylnonacosanes | branched alkane | 408 | 36.359 | 2931 | 0.3 ± 0.1 | 0.31 ± 0.05 | 0.3 ± 0.1 | 0.4 ± 0.1 | 0.72 ± 0.01 | 0.4 ± 0.1 | 0.7 ± 0.1 | 0.7 ± 0.1 |
| 32 | n-triacontane | alkane | 422 | 37.259 | 3000 | 0.02 ± 0.01 | 0.014 ± 0.003 | 0 ± 0 | 0.015 ± 0.005 | 0.043 ± 0.004 | 0.021 ± 0.003 | 0.04 ± 0.01 | 0.04 ± 0.01 |
| 33 | Hentriacontadiene | alkadiene | 418 | 38.069 | 3064 | 0.015 ± 0.004 | 0.013 ± 0.004 | 0.01 ± 0 | 0.02 ± 0.01 | 0.05 ± 0.01 | 0.013 ± 0.005 | 0.04 ± 0.02 | 0.058 ± 0.004 |
| 34 | 9-hentriacontene | alkene | 420 | 38.241 | 3077 | 0.7 ± 0.2 | 0.6 ± 0.1 | 0.5 ± 0.1 | 0.4 ± 0.1 | 1 ± 0.1 | 0.44 ± 0.02 | 1.2 ± 0.3 | 0.9 ± 0.1 |
| 35 | 7-hentriacontene | alkene | 420 | 38.328 | 3084 | 0.8 ± 0.2 | 0.5 ± 0.1 | 0.4 ± 0.1 | 0.3 ± 0.1 | 0.9 ± 0.1 | 0.46 ± 0.04 | 1.4 ± 0.3 | 0.8 ± 0.1 |
| 36 | n-hentriacontane | alkane | 436 | 38.533 | 3100 | 0.6 ± 0.1 | 0.5 ± 0.1 | 0.5 ± 0.1 | 0.4 ± 0.1 | 1.1 ± 0.1 | 0.5 ± 0.1 | 1.5 ± 0.4 | 1.2 ± 0.2 |
| 37 | Methylhentriacontane | branched alkane | 436 | 38.901 | 3115 | 0.05 ± 0.01 | 0.05 ± 0.01 | 0.04 ± 0.01 | 0.05 ± 0.01 | 0.19 ± 0.02 | 0.086 ± 0.019 | 0.16 ± 0.04 | 0.18 ± 0.03 |
| 38 | Tritriacontadiene | alkadiene | 436 | 40.496 | 3179 | 0.01 ± 0.01 | 0.0067 ± 0.0004 | 0.02 ± 0 | 0.03 ± 0.01 | 0.05 ± 0.01 | 0.013 ± 0.002 | 0.07 ± 0.02 | 0.08 ± 0.02 |
| 39 | triatriacontene^*^ | alkene | 434 | 40.738 | 3189 | 0.5 ± 0.1 | 0.5 ± 0.1 | 0.3 ± 0.1 | 0.3 ± 0.1 | 0.9 ± 0.1 | 0.3 ± 0.1 | 1.1 ± 0.2 | 1 ± 0.1 |
| 40 | n-tritriacontane | alkane | 464 | 41.015 | 3300 | 0.016 ± 0.005 | 0.02 ± 0.01 | 0.001 ± 0.001 | 0 ± 0 | 0.08 ± 0.01 | 0.005 ± 0.002 | 0.07 ± 0.02 | 0.06 ± 0.02 |
|  |  |  |  |  |  |  |  |  |  |  |  |  |  |
|  |  | alkanes |  |  |  | 64 ± 5 | 75 ± 3 | 58 ± 11 | 76 ± 6 | 101 ± 4 | 73 ± 6 | 66 ± 8 | 89 ± 6 |
|  |  | alkenes |  |  |  | 10 ± 1 | 10.4 ± 0.7 | 8 ± 2 | 13 ± 1 | 20 ± 2 | 10 ± 1 | 12 ± 1 | 17 ± 1 |
|  |  | alkadienes |  |  |  | 0.4 ± 0.1 | 0.4 ± 0.1 | 0.4 ± 0.1 | 0.52 ± 0.03 | 0.4 ± 0.1 | 0.4 ± 0.1 | 0.8 ± 0.2 | 0.7 ± 0.1 |
|  |  | branched alkanes |  |  |  | 1.5 ± 0.3 | 1.6 ± 0.2 | 1.5 ± 0.4 | 1.9 ± 0.4 | 3.4 ± 0.1 | 2 ± 0.4 | 2.5 ± 0.3 | 2.8 ± 0.1 |
|  |  | Total^#^ |  |  |  | 78 ± 6 | 89 ± 4 | 72 ± 14 | 96 ± 8 | 134 ± 6 | 87 ± 8 | 86 ± 10 | 114 ± 8 |

^*^ Isomers of triacontane (unknown double bond position).

^ξ^ NI: not-identified compound

^ⱡ^ UK: unknown CHC group

^#^ Total including NI
