# Supplementary material for: Sub-lethal effects of the consumption of Eupatorium buniifolium essential oil in honeybees
Source: PLoS One. 2020 Nov 4;15(11):e0241666. doi: 10.1371/journal.pone.0241666 (PMC7641371; doi:10.1371/journal.pone.0241666)
Supplement: S2 Table — 2 EOEb were supplied: EOEb-2009 and EOEb-2014). Results are shown as mean ± se (N = 5 per treatment). (DOCX) [file pone.0241666.s003.docx]

**S2 Table: Amount of CHC in honeybees with different health status** (healthy and *Nosema*-infected; Experiment II), fed on different diets (EOEB-supplemented and control diets. 2 EOEb were supplied: EOEb-2009 and EOEb-2014) and. Results are shown as mean ± se (N = 5 per treatment).

|  | |  | |  | | **Amount of CHC (μg/bee)** | | | | | | | |
| --- | --- | --- | --- | --- | --- | --- | --- | --- | --- | --- | --- | --- | --- |
| **Peak N^o^** | **Compound ID** | **Compound group** | **MW** | **Retention Time** | **Retention index** | **Control** | **Control (Ethanol)** | **Control + Nos.** | **EOEb_2014+Nos.** | **EOEb_2014** | **EOEb_2009** | **EOEb_2009+Nos.** | **Ethanol+Nos.** |
| 2 | NI^ξ^ | UK^ⱡ^ |  | 12.138 | 1476 | 0.3 ± 0.1 | 0.2 ± 0.1 | 0.2 ± 0.1 | 7.4 ± 1.2 | 3.1 ± 1 | 4.6 ± 0.7 | 3.6 ± 0.4 | 0.3 ± 0.1 |
| 3 | NI | UK |  | 12.96 | 1521 | 0.3 ± 0.1 | 0.26 ± 0.05 | 0.33 ± 0.21 | 1.83 ± 0.53 | 0.95 ± 0.33 | 1.69 ± 0.24 | 1.44 ± 0.29 | 0.2 ± 0.06 |
| 4 | NI | UK |  | 15.994 | 1687 | 0.4 ± 0.1 | 0.29 ± 0.04 | 0.3 ± 0.1 | 0.14 ± 0.02 | 0.2 ± 0.1 | 0.4 ± 0.1 | 0.4 ± 0.1 | 0.4 ± 0.1 |
| 5 | n-nonadecane | alkane | 268 | 19.874 | 1900 | 3.9 ± 0.6 | 1.9 ± 0.3 | 1.3 ± 0.4 | 1.3 ± 0.2 | 1.4 ± 0.4 | 2.3 ± 0.4 | 1.7 ± 0.4 | 1.8 ± 0.5 |
| 6 | NI | UK |  | 20.351 | 1937 | 0.07 ± 0.01 | 0.06 ± 0.02 | 0.1 ± 0.1 | 0.1 ± 0.03 | 0.07 ± 0.03 | 0.1 ± 0.03 | 0.05 ± 0.01 | 0.07 ± 0.01 |
| 7 | n-eicosane | alkane | 282 | 21.147 | 2000 | 0.14 ± 0.04 | 0.1 ± 0.03 | 0.1 ± 0.1 | 0.12 ± 0.04 | 0.1 ± 0.1 | 0.2 ± 0.1 | 0.1 ± 0.1 | 0.12 ± 0.02 |
| 8 | n-heneicosane | alkane | 296 | 23.572 | 2100 | 4.9 ± 0.7 | 4.1 ± 0.4 | 2 ± 0.3 | 3 ± 0.3 | 3.3 ± 0.9 | 5.2 ± 0.7 | 3.3 ± 0.5 | 3 ± 0.7 |
| 9 | n-docosane | alkane | 310 | 25.324 | 2200 | 0.6 ± 0.2 | 0.7 ± 0.2 | 0.28 ± 0.03 | 0.7 ± 0.1 | 0.4 ± 0.1 | 0.9 ± 0.2 | 0.6 ± 0.1 | 0.4 ± 0.1 |
| 10 | tricosadiene | alkadiene | 306 | 26.542 | 2272 | 0.2 ± 0.1 | 0.13 ± 0.04 | 0.1 ± 0.02 | 0.2 ± 0.1 | 0.2 ± 0.1 | 0.3 ± 0.1 | 0.4 ± 0.1 | 0.2 ± 0.1 |
| 11 | 9-tricosene | alkene | 308 | 26.589 | 2274 | 2.6 ± 0.8 | 5.1 ± 2.7 | 1.7 ± 0.2 | 2.7 ± 0.5 | 1.3 ± 0.5 | 3.7 ± 0.7 | 3.7 ± 0.9 | 2.4 ± 0.6 |
| 12 | 7-tricosene | alkene | 308 | 26.704 | 2281 | 0.3 ± 0.1 | 0.4 ± 0.1 | 0.22 ± 0.04 | 0.28 ± 0.01 | 0.3 ± 0.1 | 0.7 ± 0.2 | 0.5 ± 0.1 | 0.3 ± 0.1 |
| 13 | n-tricosane | alkane | 324 | 27.023 | 2300 | 17.2 ± 2.2 | 17.4 ± 2.2 | 15.2 ± 1 | 20.5 ± 1.6 | 12.5 ± 1.9 | 22.6 ± 2.6 | 23.1 ± 2.5 | 17 ± 2.8 |
| 14 | n-tetracosane | alkane | 338 | 28.643 | 2400 | 1.4 ± 0.5 | 1.1 ± 0.1 | 0.9 ± 0.1 | 1.5 ± 0.1 | 0.8 ± 0.3 | 2.2 ± 0.6 | 1.8 ± 0.3 | 1.3 ± 0.3 |
| 15 | Pentacosadiene | alkadiene | 334 | 29.775 | 2472 | 0.7 ± 0.3 | 0.6 ± 0.2 | 0.6 ± 0.1 | 0.7 ± 0.1 | 0.4 ± 0.1 | 1.3 ± 0.4 | 1.9 ± 0.5 | 0.8 ± 0.3 |
| 16 | 9-pentacosene | alkene | 336 | 29.831 | 2475 | 4.1 ± 1.1 | 4.6 ± 0.7 | 3.6 ± 0.5 | 5.3 ± 0.6 | 2 ± 0.8 | 5.8 ± 0.9 | 6.1 ± 1.1 | 4.6 ± 1 |
| 17 | 7-pentacosene | alkene | 336 | 29.945 | 2482 | 1.5 ± 0.4 | 1.4 ± 0.2 | 1.6 ± 0.4 | 2.2 ± 0.1 | 0.8 ± 0.3 | 2.3 ± 0.4 | 2.6 ± 0.6 | 2 ± 0.5 |
| 18 | n-pentacosane | alkane | 352 | 30.223 | 2500 | 19.3 ± 2.7 | 21.3 ± 1.9 | 22 ± 2.1 | 28.3 ± 1.6 | 13.9 ± 2.9 | 28.4 ± 3 | 29 ± 3.4 | 23.8 ± 2.9 |
| 19 | Methylpentacosanes | branched alkane | 352 | 30.736 | 2534 | 1.3 ± 0.5 | 1 ± 0.2 | 0.8 ± 0.2 | 1.8 ± 0.3 | 0.6 ± 0.2 | 1.7 ± 0.4 | 1.1 ± 0.3 | 1.1 ± 0.3 |
| 20 | n-hexacosane | alkane | 366 | 31.724 | 2600 | 1.5 ± 0.6 | 1.3 ± 0.2 | 1.2 ± 0.1 | 1.7 ± 0.1 | 0.7 ± 0.3 | 2.6 ± 0.8 | 2.1 ± 0.4 | 1.7 ± 0.5 |
| 21 | Heptacosadiene | alkadiene | 362 | 32.643 | 2662 | 0.2 ± 0.1 | 0.18 ± 0.03 | 0.1 ± 0.1 | 0.3 ± 0.1 | 0.07 ± 0.03 | 0.12 ± 0.05 | 0.08 ± 0.02 | 0.11 ± 0.03 |
| 22 | 9-heptacosene | alkene | 364 | 32.844 | 2676 | 2.7 ± 1.3 | 2.9 ± 0.5 | 2.2 ± 0.2 | 3.1 ± 0.4 | 1.3 ± 0.5 | 4.5 ± 1.3 | 4.6 ± 1 | 3.2 ± 0.8 |
| 23 | 7-heptacosene | alkene | 364 | 32.955 | 2684 | 0.8 ± 0.4 | 0.8 ± 0.1 | 0.7 ± 0.1 | 1 ± 0.1 | 0.5 ± 0.2 | 1.5 ± 0.5 | 1.3 ± 0.2 | 1.1 ± 0.3 |
| 24 | n-heptacosane | alkane | 380 | 33.195 | 2700 | 20.2 ± 2.3 | 22.9 ± 1.9 | 22.4 ± 1.3 | 26.9 ± 2 | 12.8 ± 2.7 | 28.5 ± 3.7 | 26.8 ± 2.8 | 24.8 ± 3.6 |
| 25 | Methylhetptacosanes | branched alkane | 380 | 33.641 | 2732 | 5.7 ± 1.6 | 4.3 ± 0.6 | 4 ± 0.8 | 6.8 ± 1.1 | 2.7 ± 0.9 | 6.8 ± 1.4 | 4.9 ± 0.9 | 5 ± 1.2 |
| 26 | n-octacosane | alkane | 394 | 34.586 | 2800 | 0.9 ± 0.4 | 0.6 ± 0.1 | 0.5 ± 0.1 | 0.9 ± 0.1 | 0.3 ± 0.1 | 1.3 ± 0.6 | 0.9 ± 0.2 | 0.7 ± 0.2 |
| 27 | nonacosadiene? | alkadiene | 390 | 35.444 | 2863 | 2 ± 1 | 2.2 ± 0.4 | 1.5 ± 0.6 | 2.2 ± 0.7 | 0.9 ± 0.4 | 3.4 ± 1.2 | 2.5 ± 0.5 | 1.5 ± 0.4 |
| 28 | 9-nonacosene | alkene | 406 | 35.652 | 2878 | 1.7 ± 0.7 | 2.1 ± 0.3 | 1.3 ± 0.1 | 1.9 ± 0.5 | 0.9 ± 0.3 | 3.6 ± 1.1 | 2.9 ± 0.8 | 2.2 ± 0.6 |
| 29 | 7-nonacosene | alkene | 406 | 35.708 | 2882 | 1.1 ± 0.4 | 0.8 ± 0.1 | 0.7 ± 0.2 | 0.8 ± 0.1 | 0.4 ± 0.2 | 1.4 ± 0.3 | 1.1 ± 0.2 | 0.9 ± 0.2 |
| 30 | n-nonacosane | alkane | 408 | 35.952 | 2900 | 9.9 ± 1.7 | 9 ± 1.1 | 8.5 ± 0.8 | 10.6 ± 1 | 4.9 ± 1.4 | 13.4 ± 2.2 | 11.3 ± 1.4 | 10.4 ± 2.1 |
| 31 | Methylnonacosanes | branched alkane | 408 | 36.359 | 2931 | 3.2 ± 1 | 2.4 ± 0.3 | 2.1 ± 0.4 | 3.6 ± 0.6 | 1.4 ± 0.5 | 3.9 ± 1 | 3.1 ± 0.6 | 2.8 ± 0.7 |
| 32 | n-triacontane | alkane | 422 | 37.259 | 3000 | 0.3 ± 0.1 | 0.16 ± 0.03 | 0.12 ± 0.01 | 0.2 ± 0.1 | 0.1 ± 0.04 | 0.5 ± 0.2 | 0.27 ± 0.03 | 0.2 ± 0.1 |
| 33 | Hentriacontadiene | alkadiene | 418 | 38.069 | 3064 | 0.2 ± 0.1 | 0.2 ± 0.04 | 0.15 ± 0.01 | 0.16 ± 0.04 | 0.4 ± 0.3 | 0.3 ± 0.1 | 0.22 ± 0.05 | 0.2 ± 0.1 |
| 34 | 9-hentriacontene | alkene | 420 | 38.241 | 3077 | 3.6 ± 0.7 | 2.9 ± 0.5 | 2.4 ± 0.3 | 2.6 ± 0.4 | 1.8 ± 0.6 | 4.8 ± 1.1 | 3.4 ± 0.3 | 3.2 ± 0.9 |
| 35 | 7-hentriacontene | alkene | 420 | 38.328 | 3084 | 3.4 ± 0.7 | 2.6 ± 0.4 | 2.3 ± 0.4 | 2.6 ± 0.4 | 1.3 ± 0.6 | 4.8 ± 1.1 | 2.9 ± 0.5 | 3.2 ± 0.8 |
| 36 | n-hentriacontane | alkane | 436 | 38.533 | 3100 | 4.6 ± 1.2 | 3.9 ± 0.7 | 3.8 ± 0.3 | 4.4 ± 0.9 | 3.2 ± 1.7 | 6.6 ± 1.7 | 5.9 ± 1 | 5.5 ± 1.4 |
| 37 | Methylhentriacontane | branched alkane | 436 | 38.901 | 3115 | 1 ± 0.4 | 0.6 ± 0.1 | 0.7 ± 0.1 | 1 ± 0.2 | 0.6 ± 0.3 | 1.2 ± 0.3 | 1 ± 0.3 | 0.8 ± 0.2 |
| 38 | Tritriacontadiene | alkadiene | 436 | 40.496 | 3179 | 0.6 ± 0.2 | 0.5 ± 0.1 | 0.5 ± 0.1 | 0.6 ± 0.2 | 0.4 ± 0.2 | 0.8 ± 0.2 | 0.86 ± 0.03 | 0.9 ± 0.3 |
| 39 | tritriacontene^*^ | alkene | 434 | 40.738 | 3189 | 6.1 ± 2.1 | 5.5 ± 0.8 | 4.7 ± 0.8 | 6.8 ± 1.4 | 3.6 ± 1.2 | 10.5 ± 2.7 | 8.5 ± 1.8 | 7.1 ± 1.7 |
| 40 | n-tritriacontane | alkane | 464 | 41.015 | 3300 | 0.9 ± 0.5 | 0.3 ± 0.1 | 0.2 ± 0.1 | 0.3 ± 0.1 | 0.6 ± 0.4 | 0.7 ± 0.2 | 0.8 ± 0.3 | 0.5 ± 0.1 |
|  |  |  |  |  |  |  |  |  |  |  |  |  |  |
|  |  | alkane |  |  |  | 85.7 ± 12.7 | 68 ± 18 | 78.6 ± 5.8 | 100.3 ± 6.8 | 55 ± 11.9 | 115.3 ± 15.3 | 107.6 ± 12.6 | 91.3 ± 13.9 |
|  |  | alkene |  |  |  | 27.9 ± 8.6 | 21.9 ± 6.1 | 21.6 ± 2.5 | 29.1 ± 4 | 14.2 ± 5.3 | 43.6 ± 9.2 | 37.7 ± 7.2 | 30.2 ± 7.1 |
|  |  | alkadiene |  |  |  | 3.8 ± 1.6 | 3 ± 0.9 | 3.1 ± 0.7 | 4.1 ± 1 | 2.5 ± 0.8 | 6.2 ± 1.9 | 6.1 ± 1.1 | 3.8 ± 1 |
|  |  | branched alkane |  |  |  | 11.2 ± 3.4 | 6.8 ± 2 | 7.6 ± 1.3 | 13.1 ± 2.2 | 5.4 ± 2 | 13.6 ± 3.1 | 10.1 ± 2.1 | 9.7 ± 2.4 |
|  |  | Total^#^ |  |  |  | 129.7 ± 26.3 | 100.5 ± 26.9 | 111.8 ± 10.5 | 156.1 ± 15.4 | 81.3 ± 20.8 | 185.6 ± 29.5 | 166.9 ± 23.4 | 135.9 ± 24.2 |

^*^ Isomers of triacontane (unknown double bond position).

^ⱡ^ NI: not-identified compound

^ⱡ^ UK: unknown CHC group

^#^ Total including NI
