# Supplementary material for: Sub-lethal effects of the consumption of Eupatorium buniifolium essential oil in honeybees
Source: PLoS One. 2020 Nov 4;15(11):e0241666. doi: 10.1371/journal.pone.0241666 (PMC7641371; doi:10.1371/journal.pone.0241666)
Supplement: S3 Table — Analyses were run in the Metaboanalyst platform [44]. NI: non identified CHC, UK: unknowns -sum of NI-. (DOCX) [file pone.0241666.s004.docx]

**S3 Table: Comparison between individual CHC from control and control (ethanol) honeybees** (univariate analysis results, P-values of the individual t-tests, no significant differences were found). Analyses were run in the Metaboanalyst platform (1). (NI: non identified CHC, UK: unknowns (sum of NIs).)

|  | **p-value** | |
| --- | --- | --- |
| **Compound** | **Experiment I** | **Experiment II** |
| NI | 0.75 | 0.23 |
| NI | 0.41 | 0.96 |
| NI | 0.47 | 0.31 |
| n-nonadecane | 0.57 | 0.03 |
| NI | 0.27 | 0.20 |
| n-eicosane | 0.27 | 0.32 |
| n-heneicosane | 0.46 | 0.37 |
| n-docosane | 0.69 | 0.64 |
| tricosadiene | 0.83 | 0.65 |
| 9-tricosene | 0.05 | 0.47 |
| 7-tricosene | 0.49 | 0.38 |
| n-tricosane | 0.17 | 0.97 |
| n-tetracosane | 0.53 | 0.86 |
| pentacosadiene | 0.57 | 0.85 |
| 9-pentacosene | 0.14 | 0.52 |
| 7-pentacosene | 0.14 | 0.97 |
| n-pentacosane | 0.20 | 0.46 |
| methylpentacosanes | 0.89 | 0.57 |
| n-hexacosane | 0.50 | 0.89 |
| heptacosadiene | 0.49 | 0.89 |
| 9-heptacosene | 0.33 | 0.44 |
| 7-heptacosene | 0.64 | 0.54 |
| n-heptacosane | 0.57 | 0.39 |
| methylhetptacosanes | 0.77 | 0.44 |
| n-octacosane | 0.31 | 0.68 |
| nonacosadiene | 0.89 | 0.41 |
| 9-nonacosene | 0.89 | 0.27 |
| 7-nonacosene | 0.27 | 0.82 |
| n-nonacosane | 0.19 | 0.79 |
| methylnonacosanes | 0.97 | 0.62 |
| n-triacontane | 0.55 | 0.70 |
| hentriacontadiene | 0.78 | 0.57 |
| 9-hentriacontene | 0.52 | 0.57 |
| 7-hentriacontene | 0.25 | 0.35 |
| n-hentriacontane | 0.57 | 0.76 |
| methylhentriacontanes | 0.88 | 0.42 |
| tritriacontadiene | 0.49 | 0.69 |
| tritriacontene | 0.90 | 0.90 |
| n-tritriacontane | 0.55 | 0.24 |
| Alkanes | 0.13 | 0.90 |
| Alkadienes | 0.77 | 0.90 |
| Alkene | 0.74 | 0.90 |
| Branched alkanes | 0.82 | 0.55 |
| UK | 0.80 | 0.90 |
