# Supplementary material for: Sub-lethal effects of the consumption of Eupatorium buniifolium essential oil in honeybees
Source: PLoS One. 2020 Nov 4;15(11):e0241666. doi: 10.1371/journal.pone.0241666 (PMC7641371; doi:10.1371/journal.pone.0241666)
Supplement: S4 Table — (DOCX) [file pone.0241666.s005.docx]

**S4 Table: Comparisons of the individual 39 CHC in healthy vs. *Nosema*-infected bees** (2-factor-ANOVA, significant differences in bold).

| **Compounds** | | **p-values for factors** | |
| --- | --- | --- | --- |
| **No** | **Compound** | **Product** | **Health** |
| 4 | n-nonadecane | 0.073 | **0.021** |
| 6 | n-eicosane | 0.85 | 0.864 |
| 7 | n-heneicosane | 0.598 | **0.004** |
| 8 | n-docosane | 0.623 | 0.289 |
| 9 | tricosadiene | 0.168 | 0.503 |
| 10 | 9-tricosene | 0.366 | 0.511 |
| 11 | 7-tricosene | 0.07 | 0.437 |
| 12 | n-tricosane | 0.09 | 0.331 |
| 13 | n-tetracosane | 0.192 | 0.776 |
| 14 | pentacosadiene | 0.1 | 0.125 |
| 15 | 9-pentacosene | 0.146 | 0.213 |
| 16 | 7-pentacosene | 0.129 | **0.024** |
| 17 | n-pentacosane | 0.07 | **0.014** |
| 18 | methylpentacosanes | 0.859 | 0.772 |
| 19 | n-hexacosane | 0.18 | 0.51 |
| 20 | heptacosadiene | 0.55 | 0.73 |
| 21 | 9-heptacosene | 0.16 | 0.14 |
| 22 | 7-heptacosene | 0.296 | 0.195 |
| 23 | n-heptacosane | 0.079 | **0.048** |
| 24 | methylhetptacosaneSs | 0.793 | 0.607 |
| 25 | n-octacosane | 0.47 | 0.787 |
| 26 | nonacosadiene | 0.54 | 0.996 |
| 27 | 9-nonacosene | 0.051 | 0.56 |
| 28 | 7-nonacosene | 0.161 | 0.853 |
| 29 | n-nonacosane | 0.099 | 0.395 |
| 30 | methylnonacosane | 0.601 | 0.603 |
| 31 | n-triacontane | 0.286 | 0.758 |
| 32 | hentriacontadiene | 0.751 | 0.554 |
| 33 | 9-hentriacontene | 0.16 | 0.608 |
| 34 | 7-hentriacontene | 0.109 | 0.806 |
| 35 | n-hentriacontane | 0.444 | 0.17 |
| 36 | methylhentriacontane | 0.704 | 0.736 |
| 37 | tritriacontadiene | 0.515 | 0.129 |
| 38 | X-tritriacontene | 0.161 | 0.597 |
| 39 | n-tritriacontane | 0.759 | 0.415 |

1. Chong J, Wishart DS, Xia J. Using metaboanalyst 4.0 for comprehensive and integrative metabolomics data analysis. Current protocols in bioinformatics. 2019;68(1):e86.
