# Supplementary material for: Sub-lethal effects of the consumption of Eupatorium buniifolium essential oil in honeybees
Source: PLoS One. 2020 Nov 4;15(11):e0241666. doi: 10.1371/journal.pone.0241666 (PMC7641371; doi:10.1371/journal.pone.0241666)
Supplement: Attachment — Submitted filename: Response to reviewers round 2.docx [file pone.0241666.s006.docx]

Montevideo, October 16, 2020

Response to reviewers (also included in the cover letter)

Comments from Reviewer #1 and answers:

1) *Upon reading the revised version, I found L 30-38 (in abstract) reads circuitous and they must be re-organized. Also the abstract should be one merged paragraph instead of several separate ones.*

Answer:

Abstract was edited. L 30-38 are now changed.

The abstract is now one paragraph

2) *Moreover, authors are suggested to be careful with the format requited by the journal; for example, I have see several small paragraphs in the “Statistical analyses” part. After revisions, I think the new version will be suitable for publishing in the journal.*

Answer:

“Statistical analyses” part was re-written.

The whole MS was also checked to fix format flaws.

Reviewer #2 did not make any further suggestion.

Carmen Rossini, Ph.D.

Co-chair of *Laboratorio de Ecología Química, Facultad de Química*, UdelaR-Uruguay

[crossini@fq.edu.uy](mailto:crossini@fq.edu.uy)

[crossinister@gmail.com](mailto:crossinister@gmail.com)
